# The Construction of Biological ‘Inter-Identity’ as the Outcome of a Complex Process of Protocell Development in Prebiotic Evolution

**DOI:** 10.3389/fphys.2020.00530

**Published:** 2020-05-26

**Authors:** Kepa Ruiz-Mirazo, Ben Shirt-Ediss, Miguel Escribano-Cabeza, Alvaro Moreno

**Affiliations:** ^1^Department of Logic and Philosophy of Science, University of the Basque Country, San Sebastian, Spain; ^2^Biofisika Institute (CSIC, UPV-EHU), Leioa, Spain; ^3^Interdisciplinary Computing and Complex BioSystems Group, Newcastle University, Newcastle upon Tyne, United Kingdom

**Keywords:** origins of life, prebiotic systems chemistry, reproducing protocells, pre-Darwinian evolution, minimal autonomy, ecopoiesis, LUCA

## Abstract

The concept of identity is used both (i) to distinguish a system as a particular material entity that is conserved as such in a given environment (token-identity: i.e., identity as permanence or endurance over time), and (ii) to relate a system with other members of a set (type-identity: i.e., identity as an equivalence relationship). Biological systems are characterized, in a minimal and universal sense, by a highly complex and dynamic, far-from-equilibrium organization of very diverse molecular components and transformation processes (i.e., ‘genetically instructed cellular metabolisms’) that maintain themselves in constant interaction with their corresponding environments, including other systems of similar nature. More precisely, all living entities depend on a deeply convoluted organization of molecules and processes (a naturalized von Neumann constructor architecture) that subsumes, in the form of current individuals (autonomous cells), a history of ecological and evolutionary interactions (across cell populations). So one can defend, on those grounds, that living beings have an identity of their own from both approximations: (i) and (ii). These transversal and trans-generational dimensions of biological phenomena, which unfold together with the actual process of biogenesis, must be carefully considered in order to understand the intricacies and metabolic robustness of the first living cells, their underlying uniformity (i.e., their common biochemical core) and the eradication of previous –or alternative– forms of complex natural phenomena. Therefore, a comprehensive approach to the origins of life requires conjugating the actual properties of the developing complex individuals (fusing and dividing protocells, at various stages) with other, population-level features, linked to their collective-evolutionary behavior, under much wider and longer-term parameters. On these lines, we will argue that life, in its most basic sense, here on Earth or anywhere else, demands crossing a high complexity threshold and that the concept of ‘inter-identity’ can help us realize the different aspects involved in the process. The article concludes by pointing out some of the challenges ahead if we are to integrate the corresponding explanatory frameworks, physiological and evolutionary, in the hope that a more general theory of biology is on its way.

## On the Concept of ‘Inter-Identity’: Some Preliminary Ideas and Potential Insights for Biogenesis

Identity is closely related to the idea of *sameness*. This can be formalized through mathematics (e.g., set theory) and be used in a strict sense, provided that it remains in that abstract space of logical operations. However, as soon as it is applied to the real world, it becomes problematic. Philosophers have been particularly aware of those difficulties throughout history, from the ancient Greeks to contemporary metaphysicists, because the idea of identity is entrenched with two perennial problems of philosophy (Brubaker and Cooper, 2000; Juarrero, 2002): how to account for (i) permanence amidst manifest change and for (ii) unity amidst manifest diversity. Nature is, indeed, changeful and diverse. If one had to choose between Heraclitus or Parmenides these days, with all the scientific knowledge that we currently have at hand, it seems safer to opt for the former – embracing a process ontology *by default*, so to speak. Nevertheless, humans have also discovered that many material entities in the world stay the same for long periods of time, and can be treated as equivalent to many others “of the same kind.” Thus, our theories should also provide us with adequate explanations for the emergence and behavior of these stationary and repeated objects/features/patterns found in nature. For instance, think about atoms, gold atoms, to take a simple case (gold is a monoisotopic element of the periodic table). Each gold atom is highly stable and, in practice, totally equivalent to any other gold atom in the universe. Thus, it looks like the identity of gold atoms, both in terms of permanence (i.e., gold’s *token* identity) and of uniformity (i.e., gold’s *type* identity), is out of question – and this is probably one of the reasons why humans appreciate so much pieces of metal that contain many such atoms.

However, stability or equivalence relationships, in general, cannot be taken for granted. Quite the contrary: nature is intrinsically heterogeneous and variable, as we just asserted. The issue of identity becomes especially tricky when dealing with complex systems, whose maintenance (as such systems) depends on the dynamic organization and non-linear interactions among its constituent parts (in continuous renewal and/or transformation), as well as with its local environment. For instance, *dissipative structures* (e.g., Benárd convection cells or B-Z chemical waves; Prigogine, 1980) involve global, macroscopic stationary states, which remain stationary as long as a set of suitable boundary conditions keep (or are kept) constant. Furthermore, the corresponding patterns of order (which result from the convoluted fluid dynamics or inorganic reaction couplings among their numerous components, under those conditions) are equivalent each time you run the experiment, regardless of the specific moment or location when/where the phenomenon occurs. Yet, the stability of these far-from-equilibrium systems is much more precarious than quasi-equilibrium structures (e.g., self-assembled molecular aggregates), let alone atoms at equilibrium. Their identity is thus dependent on their being open systems in constant interaction with their environment: i.e., they constitute themselves *through* interaction (Collier and Hooker, 1999; Bickhard, 2000; Bishop, 2012). Besides, for systems that encompass such a large number of components (millions and millions of molecules moving or reacting with each other) one can always identify differences in details that make each pattern of order unique, of course – even if those minor differences pass disregarded when the pattern is grouped together with other, highly similar ones.

In this article, we are going to deal with the origin and evolutionary development of phenomena that are not just complex, in the previous sense, but *hyper-*complex: namely, proto-biological processes and entities. Living organisms, as we know them on planet Earth, have achieved a dynamic robustness and a capacity for long-term sustainability that is really striking from the point of view of fundamental physics. When far-from-equilibrium phenomena occur in the inert domain, they consistently tend to degrade and disappear – quite sooner than later, in fact. In contrast, life persists on the planet for thousands of millions of years, as a cyclic, recurrent and collective phenomenon, that projects far beyond the individual units that temporarily instantiate its most characteristic features, like metabolism or adaptive agency. Although the biosphere at present consists of an extremely rich variety of living forms, both unicellular and multicellular, we have discovered, over the years, an amazing unity behind all this variety. Summing up the work of a few generations of molecular biologists and biochemists, we know now that a collection of basic mechanisms, properties and dynamic operations underlie the workings of any cell: among others, a common set of metabolic intermediaries and core metabolic pathways, shared bioenergetic principles, or a universal genetic code (Alberts et al., 2002; Stryer et al., 2015; Nelson and Cox, 2017).

This underlying unity not only gives solid support to Darwin’s key intuition that all terrestrial life comes from a single origin, through descend with modification, but suggests a population of cells of very similar characteristics as the departure point for open-ended, biological evolution and for the subsequent process of diversification and complexification of the living world, as we came to know it much later on. The apparent homogeneity of LUCA (the last universal common ancestor or *cenancestor* of all life on Earth), together with the lack of traces of previous stages (that is, prebiotic systems of intermediate complexity), indicate that a highly successful and rapidly proliferating type of organization (a community of genetically instructed cellular metabolisms) surely took over in the context of previous populations of more diverse and rudimentary protocells. That first population of living cells was strongly communal, performing massive horizontal gene transfer processes (Woese, 1998), which also contributed to their homogeneity and helped them thrive and expand in those early stages, to eventually colonize –and transform irreversibly– the surface of the whole planet.

In the following pages, we will argue that the hypothetical prokaryotic entities comprising such a community of cells, precursor of all –current and extinct– forms of life on Earth, had to constitute *identities* of a very special kind. We will further claim that any process of biogenesis, to be complete, should bring about a very similar scenario: a community of complex individuals, with tightly interconnected identities. These are *interacting* and *interactive* identities, in various complementary ways, as we will try to explain below (justifying the use of the term ‘inter-identity’ from different angles, and showing some of its most important implications). But before doing that, adopting a deeper, foundational perspective (in the first part of the article), we will make the effort to show that the individuals of this community (the first, full-fledged, minimal living organisms) indeed realize their identity both: (i) as material systems/organizations that distinguish themselves from other systems/organizations, staying the same individual that each of them was, previously in time (i.e., they constitute *token* identities); and (ii) as material systems/organizations that are, for all relevant purposes, equivalent to other systems/organizations in the group, sharing the same potentialities held by any other member of that group (i.e., they constitute *type* identities). Therefore, we will see how the ‘token/type’ dichotomy itself is somehow intermingled, or blurred, by means of real systems, living cells, that challenge such an epistemic distinction and merge, somehow, the domain of the *actual* (i.e., the regime of causal relationships at a given time, for any given individual in interaction with its environment) with the domain of the *potential* (the dynamic propensities inferred for subsequent temporal stages, pertinent to a population, group or sub-group of individuals).

Understanding how these two –almost orthogonal– dimensions of the concept of identity become, in fact, compatible will require performing an analysis at different spatial and temporal scales, from the developing protocellular systems to the level of populations evolving across generations. As elaborated below, a combination of insights coming from these different levels of description, whose integration is challenging but seldom addressed in the literature, will help us show that only *reliably reproducing* protocell organizations (i.e., *tokens* that consistently generate *types*) are capable of realizing those two faces of identity at once. Therefore, we will reach the conclusion that in order to solve the problem of origins of life, it is not sufficient to consider minimalist autopoietic systems/organizations (compartmentalized proto-metabolisms), but one is forced to explore more complex cellular architectures (genetically instructed metabolisms) that actually subsume wider and longer term relationships within an ecologically structured and phylogenetically evolving and unfolding population of such cells.

The article is constructed in the following way. First, in section “A Plausible Departure Point: The ‘Heterogeneous Protocell Population Scenario’,” we describe a highly plausible starting point for the process of origins of life: namely, a messy, colloidal environment in which rudimentary protocells undergo fission and fusion events, triggered by non-linear chemistries linked to self-assembly and self-organization phenomena. Then, in section “A Highly Complex Final Stage: The Origin-of-Life ‘Singularity’,” we consider LUCA (i.e., a minimalist population of prokaryotic life), as equivalent to the final stage of the process and analyze its nature, both from the individual and population perspectives. The comparison between those two completely different scenarios (the starting and final stages of biogenesis) will help us explain, in more abstract terms (see section “Core Discussion: The Construction of Biological ‘Inter-identity’ as the Outcome of a Complex Process of Prebiotic Evolutionary Development”), how the relationship between ‘token-identities’ and ‘type-identities’ must become increasingly tighter and interwoven throughout prebiotic evolution. This will lead us to propose the main thesis of this work: biogenesis can –and should– be conceived as a process of evolutionary development of increasingly complex protocells until they accomplish biological ‘inter-identity,’ eliminating the previous, more precarious and diverse populations of interacting individuals. Finally, in the last section, we make some more general concluding remarks about the importance of keeping a genealogical perspective in the natural sciences (i.e., of addressing seriously the problem of origins of life) in order to understand the main principles on which a coherent theory of *evolutionary systems biology* should be founded.

## A Plausible Departure Point: the ‘Heterogeneous Protocell Population Scenario’

Decades of research efforts by highly talented prebiotic chemists with the aim to discover minimal systems of self-replicating molecules (RNA oligonucleotides in particular, but also peptides or other chemical species of biological relevance – for an extensive review, see: Ruiz-Mirazo et al., 2014) have led to interesting but, overall, remarkably modest results. Probably the strong reductionist assumptions and the oversimplifications made by the majority of researchers working in the field of origins of life, under the enormous influence of molecular biology and traditional synthetic organic chemistry, hold a good part of the responsibility for such a failure. Indeed, although diverse material structures (e.g., nucleic acids) have template properties, which directly contribute to their multiplication (including the conservation of their characteristic monomeric sequences, through complementary base-pairing, during the copy process) there are not truly ‘self-replicating’ molecules anywhere in the biological sphere. Cells faithfully replicate some of their material structures, of course, but always making use of additional functional machinery. Thus, we should consider the possibility that our idealizations have been pushing the investigation about origins of life in unrealistic directions, and alternative work assumptions must definitely be explored. As one of the current leaders of the field acknowledges (Sutherland, 2017), present accomplishments have reached, at most, the end of the very beginning of the process of biogenesis: namely, the synthesis, in good yields, of the various molecular building blocks to start the process. Accordingly, the origin-of-life research community is looking forward to new experimental insights from the flourishing area of ‘systems chemistry,’ which deals with complex mixtures of molecules and their emergent properties, as the awareness about the irreducibility of biological behavior to single types of molecules, or molecular mechanisms, continues to spread across the scientific community (Kroiss et al., 2019; Ruiz-Mirazo, 2019).

**FIGURE 1:**
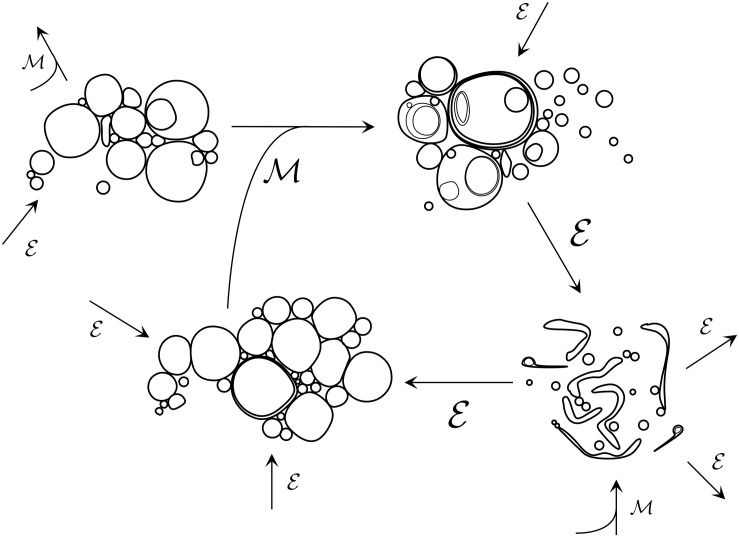
Early prebiotic chemistry would have likely entailed diverse populations of low complexity colloidal systems, engaging in myriads of aggregative/competitive interactions and transformations. Environmental events ε such as changes in external osmolarity, pH, ionic concentrations, temperature, and local fluid flow conditions would drive fluctuations in the composition of populations and could also scaffold primitive division cycles. In parallel, population mixing events ℳ would act to re-distribute and combine individuals from different populations via fusion. At this ‘messy’ stage of protocellular development, lipid vesicle populations would likely have been highly heterogeneous in terms of size, membrane composition, trapped internal molecules and lamellarity of individuals, with many individuals containing internal compartments nested in complex ways. Vesicle breakage and resealing would have been highly prevalent at this stage, too.

A much more plausible alternative prebiotic scenario, given the numerous pieces of evidence demonstrating that lipids or other *amphiphilic* compounds (molecules with both hydrophilic and hydrophobic parts) and *surfactants* (molecules that –more generally– tend to be part of water-oil or water-air interfaces) were surely present in the primitive Earth,^1^ would be a heterogeneous population of relatively simple, self-assembled protocellular systems undergoing several physical and chemical transformations. In principle, these highly dynamic protocells could consist of different kinds of supramolecular aggregates (e.g.,: micelles, vesicles, droplets, coacervates,… or, more probably, coexisting mixtures of them) but for the sake of simplicity and continuity with the biological world, we will consider here a *vesicle* suspension in water (i.e., a population of spontaneously formed spheroid compartments, containing aqueous micro-environments encapsulated by lipid bilayers – i.e., prebiotic systems already endowed with the characteristic topology of cells). However, we should not think of this as a quasi-equilibrium, homogeneously distributed suspension, in which each vesicle maintains itself as a supramolecular structure in a metastable stationary state (like standard liposome suspensions, as prepared in the lab). Proto-cellularity actually involves the coupling of self-assembly with chemical processes (Ruiz-Mirazo, 2011), favoring a much richer variety of dynamic states in out-of-equilibrium conditions. Accordingly, the actual sizes, shapes, and composition of these compartmentalized systems would be quite diverse (see Figure 1), and in continuous change, because different chemical reactions (involving other simple, prebiotically plausible molecular species, like additional amphiphiles/surfactants, aminoacids, small peptides…) would be intrinsically linked to their dynamics (Ruiz-Mirazo and Mavelli, 2008; Shirt-Ediss et al., 2014, 2015; Shirt-Ediss, 2016; Piedrafita et al., 2017), affecting both the inner aqueous core and the properties of the actual boundary (e.g., membrane permeability, fluidity, etc.).

In fact, one should not expect any simple (e.g., Poissonian-like) statistical description to be applicable to these non-equilibrium and highly heterogeneous protocell suspensions. For instance, spontaneously forming vesicles are bound to trigger cooperative/aggregative effects that lead to the uneven encapsulation of complex mixtures of organic compounds (especially if these are macromolecules – like biopolymers, as shown by Luisi’s group; Souza et al., 2009, 2011); or also more basic physical forces (e.g., osmotic imbalances across the vesicle membranes) can generate complex oscillatory behaviors in the population (as reported by other labs, like Parikh’s; Oglȩcka et al., 2014). Among the numerous transformations that these early protocellular systems could undergo (e.g., constrained diffusion and transfer of their components, membrane transient breakage and re-sealing processes, deformations, shrinkage, swelling, aggregation into clusters…), we will highlight two of special significance: *fission* and *fusion*. Fission implies the division of a vesicle into two (or more) vesicles, whereas fusion involves the merging of two (or more) vesicles into one.^2^

These transformation processes, at an early stage, need not be symmetric, nor reliably performed (in fact, they are expected to happen stochastically, involving vesicles of different sizes, shapes or compositions, and often triggered by environmental changes – see Figure 1). If that is the case (and if the general boundary conditions remain approximately constant, of course), the population of dividing and colliding protocells would not undergo any major, significant transition (in overall, statistical terms). Despite the occurrence of multiple changes in each of the individual vesicles, or the emergence of local clusters of transient complexity, or even longer-range correlations and patterns of collective dynamic behavior across wider groups of them, the protocell suspension will still look like a ‘colloidal mess,’ roughly speaking. Under those conditions, the stability of most individuals in the population (tokens) would be quite precarious: the lack of regulatory mechanisms on the growth/shrinkage dynamics of the protocells would lead, most of the times, to breakage or decay (due to osmotic imbalances or insufficient material resources in the aqueous environment) and subsequent reassembling phenomena. In turn, groups of relatively similar protocells (types – or proto-types) could be distinguished in the population, but just in terms of global, self-organization properties that would correspond to statistical patterns deriving from those ever-changing compartmentalized entities and their ongoing out-of-equilibrium dynamics, fostered by the underlying (often autocatalytic) chemistry.^3^

### The Onset of Reproductive Fission

However, the situation would radically change if fission events started to establish more consistent ‘kind correlations’ between different members of the population. In other words, if some protocellular systems developed molecular mechanisms (and a somewhat more complex organization) that enabled them to channel growth and *reproduce* themselves: i.e., if they managed to generate highly similar protocellular systems – ‘kind’ begetting ‘kind.’ This has a number of implications, which were studied in more detail through a theoretical model on the conditions for stationary reproduction of elementary protocells (Mavelli and Ruiz-Mirazo, 2013). In particular, in comparison with the initial stages (as depicted in Figure 1), protocells must involve a higher diversity of molecular components and interaction mechanisms among them (in accordance to recent laboratory experiments in which vesicle division is achieved with surprising easiness, but only provided that a number of different concurring factors are brought together; Kurisu et al., 2019). Furthermore, for the process to be recurrent, protocells must be *self-productive* in the first place, so that they can minimally control their growth dynamics and divide in such a way that the ‘offspring’ resembles the original state of the ‘mother’ protocell. By the term ‘self-productive’ here we do not mean ‘autopoietic’ in the classical or strict sense of the term (Maturana and Varela, 1980), because these protocells ought to be actually entrenched in ‘growth-division cycles’ – i.e., they would not be (highly idealized) self-maintaining entities in which a complementary relationship between metabolism and boundary ensures homeostasis, as in the original scheme (Zepik et al., 2001). Besides, these prebiotic self-productive systems should harbor a minimal degree of organizational diversity, in such a way that the same reproduction mechanisms could be realized by means of potentially different individuals.

Therefore, when we speak here about self-*re-*producing protocells we do not refer to standard vesicles or other simple compartmentalized systems, but to functionally and spatially organized reaction systems. Trivial forms of reproduction, like the multiplication of supramolecular structures, *per se*, would not lead us very far. Yet, if the protocellular system gets too complex, its reproduction will become accordingly difficult, causing a deep bottleneck (as the main results in Mavelli and Ruiz-Mirazo (2013), in fact, suggest). At this juncture, a compromise solution must be reached, in which the material organization involved is complicated enough –but not more– to be able to generate controlled cycles of growth and division (including the coordinated duplication of all of its components and transformations, their adequate spatial distribution and temporal synchronization,… so that the cycle ends up in the physical multiplication and subsequent propagation of the original organization). In other words, the appearance of protocells capable of self-reproduction, in a minimal but biologically significant sense, required protocells that had already achieved not only a certain degree of functional diversity in their organization, but also the integration of all the aforementioned processes, with mechanisms to orchestrate and modulate the necessary changes in the compartment, together with changes in the internal reaction network, in response to environmental fluctuations or stimuli (Moreno, 2019).

**FIGURE 2:**
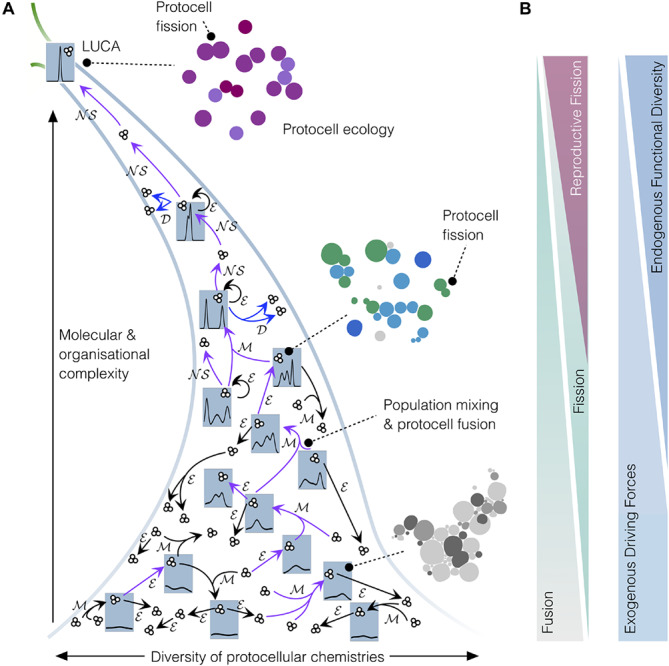
Coarse-grain analysis of the emergence of protocell *types* in populations of interacting protocells, on their journey toward LUCA. **(A)** Narrowing funnel depicts an early, ‘messy’ prebiotic chemistry with a large diversity of colloidal aggregates (individuals and groups of individuals of low molecular and organizational complexity) developing toward relatively more homogenous sets of higher-complexity individuals. Small clusters of three circles represent protocell sets/populations and blue tabs depict the probability density of protocell *types* or “families” within the whole set at each stage. At the early ‘messy’ stage, the global system did not contain identifiable types of protocells; however, mixing events ℳ would have caused recombination and fusion of individuals, sometimes resulting in individuals of higher complexity. Diverse chemistries, mixing processes, along with environmental influences ℳ would eventually lead to the emergence of the first functional protocells (purple arrows depict key transitions toward higher complexity). Once functional diversity increased sufficiently, these protocells developed the capacity to autonomously grow and reproduce (𝒟, blue arrows), starting to generate protocell types and to evolve through natural selection (𝒩𝒮), thus constituting primitive ‘lineages’ and proper ‘populations.’ **(B)** The diminishing role of environmental driving forces and protocell fusion, and conversely the increasing role of protocell fission (and, thereby, 𝒩𝒮) and functional diversification, in driving transitions toward higher complexity on the road between messy colloidal chemistry and LUCA.

At the level of the population, the transition from dynamic but globally stationary protocell organizations to *reproducing* ones will also have, of course, important and observable consequences: remarkably, much more obvious asymmetries (in terms of the underlying groups and population sub-structure) will start flourishing in the protocell suspension, since some of them will now be endowed with the intrinsic capacity to generate similar entities/organizations and, thus, potentially, to take over the whole population (or a good part of it – see Figure 2). Whether they manage to do so –or not– will depend on a number of interconnected variables (e.g., diverse growth-limiting factors, degree of stochasticity or success in the reproductive step, actual level of ‘mother-offspring’ similarity, protocell–protocell interactions…), which are usually condensed down, in classical evolutionary models, to the condition of whether their growth rate is effectively exponential or not.^4^ Anyhow, regardless of the particular interactive/competitive dynamics that may be generated among the different groups of self-(re-)productive protocells under limited availability for resources, the most relevant point here is that *tokens* start having an intrinsic potential to generate *types* in the population. And this radically changes the scene: proto-families of individuals with relatively higher similarities among them will emerge (see, again, Figure 2 – intermediate stages), since statistical/stochastic processes and other homogenizing effects can no longer compensate for those changes taking place, thanks to increasingly reliable reproduction, in specific –or at least more definite– directions within protocell ‘phenotypic space,’ so to speak.^5^

**FIGURE 3:**
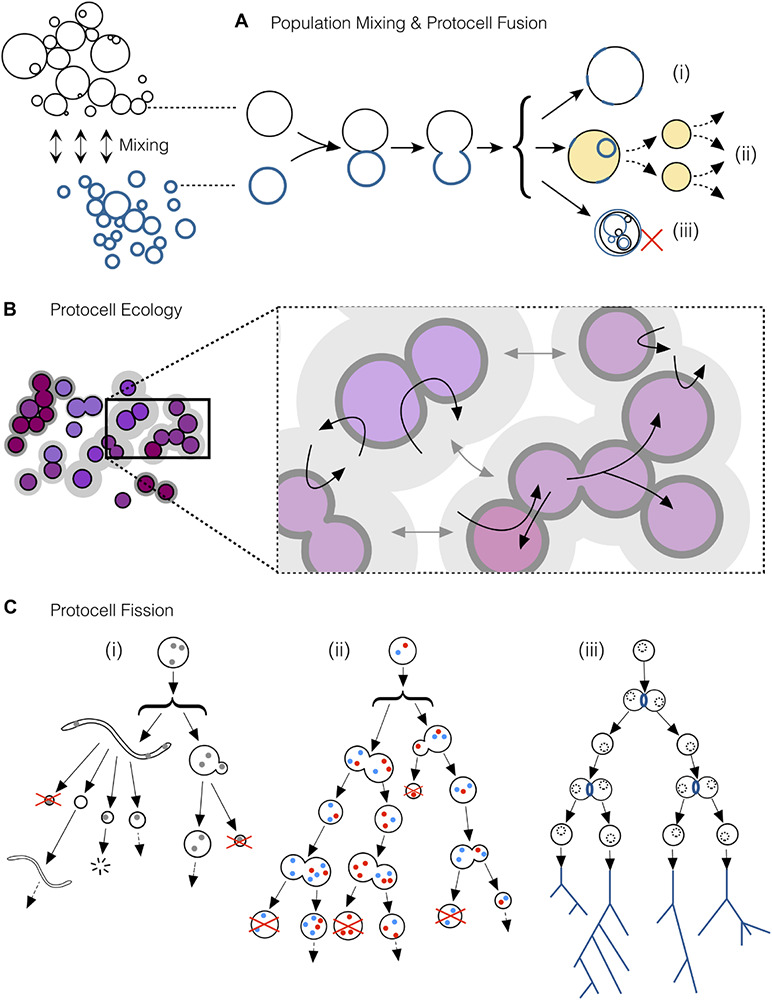
A selection of key processes driving and maintaining the emergence of protocell *types* in populations of interacting protocells. **(A)** Mixing of protocell populations, resulting in the fusion of individuals (tokens) from different populations. Protocell fusion would contribute to create (i) new functional tokens, (ii) functional tokens with the potential to become a new *type* over successive rounds of fission and (iii) novel but non-functional tokens. **(B)** *Ecopoiesis* in a population of protocells: synergetic or complementary relationships among members of different sub-populations (e.g., some protocells recycling the metabolic waste of some others) together with niche construction (due to the incipient capacities of these systems to modify their environment) would help in the recycling of limited material resources present in the environment, as well as to maintain diversity in the population composition. **(C)** Protocell fission via different pathways. **(i)** At early stages, environmental forces would scaffold the growth and division of simple protocells. **(ii)** The development of endogenous chemistries synthesizing membrane components would grant protocells the ability start dividing autonomously, but such fission would still be asymmetric and highly irregular during the first stages. Later protocells with more reliable division mechanisms would enter into “reproductive fission,” bringing about increasingly similar daughter protocells at more regular periods. When reproductive fission becomes highly reliable, as depicted in **(iii)**, natural selection gains importance, reinforcing *types* in the population and establishing the first “phylogenetic trees” (despite massive horizontal relationships and exchanges among the different protocells, not represented in this figure).

Nevertheless, the transition from bare, stochastic fission to *reproductive* fission processes in the protocell population, even if the latter become more reliable and effective with time, will not be the only aspect to be taken into account in this context. In fact, competitive interactions just based on differential reproduction of the individuals of a population, although traditionally associated with the concept of natural selection (NS), are not enough, by themselves, to develop complexity. Mechanisms to generate, manage and fix functional novelty in these prebiotic systems are also required. Otherwise, as we already argued in more detail (Moreno and Ruiz-Mirazo, 2009), the system would lead to evolutionary ‘dead ends’ (and this is particularly the case when reproduction is reduced to molecular replication). In a protocell scenario, like the one we are describing here, jumps in complexity, during initial stages, would certainly come from the non-linear couplings of self-assembling supramolecular entities, the vesicles, to more and more intricate chemistries that lead to self-production (i.e., minimal versions of compartmentalized metabolism). But these proto-metabolic systems will surely reach evolutionary bottlenecks, especially in the absence of an efficient machinery to ensure accurate heredity (i.e., the fixing and transmission of molecular and organizational features across generations). In this context, fusion events, probably preceded by vesicle aggregation phenomena (Carrara et al., 2012), will be quite critical, particularly if they involve the functional integration of those novelties previously developed in different protocells, to bring about a more complex protocellular/protometabolic organization (see Figures 2, 3 for more details).

Simon (1962) famously argued that the construction of complexity cannot be achieved in a single step. Indeed, different modules, if they come about in parallel (in our scenario: within initially independent protocells), must then be brought together in order to generate a more complex organization. This is a fundamental way of producing novelty, and fusion events should obviously contribute in that regard, but provided that the outcome of the process is adequately integrated, and the resulting (supposedly more complex) protocell remains functional (both in a metabolic and in a reproductive sense). These would be cases in which two (or more) tokens come together to generate a *novel token* which, in turn, will have (supposedly stronger) potential to spread into a new *type* in the population (see Figure 3A). Therefore, diverse interactive dynamics should come to play in the prebiotic scenario we are putting forward here: individual protocells (already belonging to incipient families/breeds) will of course compete for limited resources, and generate multiple functional variations (to be, then, selected); but this needs to be complemented with other associative or synergetic processes that are key to foster more significant jumps in complexity, when the population faces more stringent evolutionary bottlenecks. In sum, the implementation of the first major transitions in the origins of life will require that protocells develop mechanisms to achieve controlled cycles of *reproductive fission*, combined with the generation of an increasingly wider and richer domain of physiological functionalities (i.e., the protocell ‘phenotypic space’), for which –at least, occasional– events of *integrative fusion* would be also required. Nevertheless, all this will become more apparent when we describe the final stage of the process in the next section.

## A Highly Complex Final Stage: the Origin-Of-Life ‘Singularity’

All that we have learnt in biology since Darwin has confirmed the deepest insight he left for us, and for future generations: the realization that each and every species/organism inhabiting the Earth ultimately comes from the same origin. Indeed, phylogenetic studies projecting as far backwards as possible (Weiss et al., 2018), together with analyses from micropaleontology (Javaux, 2019) and comparative ‘minimal-cell’ microbiology (Xavier et al., 2014), point toward a population of unicellular prokaryotes, sharing the same basic biochemistry and a set of fundamental biomolecular mechanisms, as the end of the process of biogenesis and the beginning of biological evolution, sometime between 3.500 and 4.000 million years ago. The individuals of this population were cellular metabolisms already endowed with an intricate architecture of relationships among its molecular components and transformation processes, most prominently incarnated by a translation apparatus operating through a common genetic code.

Given the wide scope of organic chemistry (not only in terms of molecular compounds, but also reaction mechanisms, supramolecular structures, emergent dynamic behaviors, etc.), one could speculate that alternative pathways for biogenesis were surely explored by nature on the surface of the primitive Earth. Or even more bluntly, that different origins were in fact accomplished, giving birth to radically different forms of full-fledged life, which only later, after the onset of biological evolution, would have gone extinct (e.g., see Figure 3 in Javaux, 2019). However, from the evidence gathered so far, plus the application of the parsimony principle, we can just safely say that the last universal common ancestor to all forms of life, as we know them on the planet (i.e., the so-called ‘LUCA’), consisted in a population of prokaryotic cells «using nucleic acids as genetic material, 20 genetically encoded aminoacids, ribosomes for template-directed protein synthesis and membranes that allowed for chemiosmotic coupling» (Gogarten and Deamer, 2016, p. 1). There is also quite widespread consensus on the fact that horizontal gene transfer (HGT) was ubiquitous at that stage, so this original population of organisms is typically conceived as a strongly communal society of microbes, which shared many of the molecular innovations that were encoded in their collective genetic repertoire (although that repertoire also allowed for an ample variety of physiological realizations, expressed functionally/phenotypically in each cell). In contrast, the debate turns fierce when theoretical proposals attempt to go further back and postulate stages before LUCA, like the ‘progenote’ (Woese and Fox, 1977; Woese, 1998), defined as an organism (or ‘proto-organism’) in which a full-fledged genotype-phenotype relationship would be incomplete – that is, still under evolutionary development.

Nevertheless, for the purposes of this article, and once assumed (as we did in the previous section) that the most plausible starting point for biogenesis is a population of protocells (rather than a population of replicating macromolecules, like RNA) it is not really necessary to enter in the discussion about the specific features of ‘pre-LUCA’ organisms. It will be sufficient to highlight a few milestones that had to be reached during the process, and describe the general trends at work throughout it. The first aspect to underline, depicted in Figure 2 (notice the funnel), is that the chemistry in development –toward a biochemistry– implies an increase in complexity but, at the same time, a reduction of possibilities. So to speak, in order to play a more and more complex *game* of molecules, in continuous transformation, a progressively higher number of *rules* must be fixed by the emerging prebiotic systems. These specific functional rules involve a set of high-order structures and control mechanisms of diverse nature (spatial, kinetic, energetic… control mechanisms; Ruiz-Mirazo et al., 2017), which operate as enabling constraints that, thanks to their concurrent action (harnessing and coupling chemical reactions and other basic processes taking place in the system and its close surroundings), open somehow the space of possibilities for new protocell dynamic behaviors.^6^ Therefore, the most interesting and relevant restrictions in prebiotic chemistry are those that allow for –and potentially enhance– functional performance and diversification, which, among other things, are critical to establish a process of evolution by natural selection (Moreno and Ruiz-Mirazo, 2009).

Second, but not less important in order to eventually reach a ‘LUCA-like’ population, protocellular systems need to implement strategies for reproduction that are increasingly reliable, ensuring fission events in which traits are inherited with higher and higher fidelity from one generation to the other. Therefore, the actual space of functionalities in construction during these prebiotic stages is not restricted to the sphere of physiological variables or the robust maintenance of each individual, but should include those mechanisms that make reproduction more efficient, as well as the control of trans-generational variability more precise (Ruiz-Mirazo et al., 2017). In other words, the *propagation* of a given material organization (the ‘token’) becomes relevant for its *preservation*, but at a different scale (at the ‘type’ scale), which transcends the individual (see discussion in section “Core Discussion: The Construction of Biological ‘Inter-identity’ as the Outcome of a Complex Process of Prebiotic Evolutionary Development”). This trend, together with the progressive extinction of all those incipient ‘families’ or ‘breeds’ that could not withstand an increasing selective pressure, would push the protocells to be more and more similar to each other, at least at a coarse-grain level of analysis of the global outcome (see Figure 2, upper part of the funnel). Of course, taking a somewhat deeper look into the population (Figure 3), phenotypic diversity will become apparent: e.g., variations in reproduction frequency, metabolic performance and requirements (nutrient uptake, release of waste products, motility)… plus many other properties one may think of. This diversity would come from environmental variations and the stochasticity inherent to all natural phenomena, but also due to fusion events, followed by functional re-integration processes (Figure 3A) which could still be crucial to overcome evolutionary bottlenecks at those later stages, too. Furthermore, at a wider spatial and interactive scale, synchronic proto-ecological relationships among different subgroups of the global protocell population, running their metabolism on complementary sets of nutrients/waste products, would provide additional support to maintain or increase functional diversity. These primary ecological relationships (depicted in Figure 2, and in more detail in Figure 3B) would also be crucial to solve difficulties (potential extinctions or global crises) derived from resource limitations, or protocell contamination of local environments, as remarked by Guerrero (1995, 1998) (who coined the term ‘ecopoiesis’ to refer to them).

Until we elaborate the knowledge and methods required to study *in vitro* protocellular families of intermediate complexity, at the actual interface between chemistry and biology, it will be very difficult to characterize precisely the late stages of biogenesis and determine whether the process unavoidably culminates in a singularity. However, all the evidence available to date suggests that, at least when it happened on the Earth, the final ‘phase transition’ from prebiotic to biological evolution must have been remarkably narrow and uniform.^7^ Only such a singularity can explain, at the same time: first, the universality of biophysical/biochemical mechanisms supporting all forms of life on the planet; second, the huge gap between chemistry and biology, with the wiping out of other possible systems inhabiting the ‘middle-lands’ of complexity; and third, the amazing adaptive and diversification capacities of living organisms, once biological evolution got started. Indeed, this singularity must have corresponded to the successful combination of (i) a highly efficient and robust way of performing metabolism, maintaining cellular activity and organization (in non-equilibrium conditions), plus (ii) a very reliable way of propagating, in space and time, that cellular organization, transmitting (via reproduction) the material components and the architecture of relationships responsible for it to other systems.

Here the importance of generating a complex hierarchy of macro-molecular controls, articulated through a translation apparatus between nucleic acids and proteins (more precisely, between their corresponding monomeric sequences), to implement a code-mediated genotype-phenotype decoupling in each individual system cannot be overestimated. In fact, it constitutes a tremendous organizational achievement, which surely involved a prolonged co-evolutionary process (Wong, 1975; Wong et al., 2016) between biosynthetic pathways and their own products/controllers, across many protocell generations. Yet, direct and precise control on metabolism does not come for free: the controlling material structures (fundamentally, proteins) get damaged and, thus, require some turnover/repair dynamics at characteristic time scales which are –obviously– longer than the characteristic times of the controlled processes (e.g., metabolic reactions), but shorter than the lifetime of the global, cellular system where they occur. Now, the synthesis of a macro-molecule like a protein, whose functional properties depend on the specific sequence of amino acids defining its long primary structure, is not trivial at all – nor energetically cheap. This is why we do not find proteins in meteorites, or in places with no presence of living cells. But nature, ages ago, somehow managed to make the recursive construction of these highly sophisticated controllers viable, thanks to the generation of a set of quasi-inert ‘material records’ (Pattee, 1969, 1977) that operate, within these cellular systems, in a highly indirect and inactive (i.e., decoupled) way, mainly providing their template structure for transcription or replication, whenever necessary – like DNA actually does in any living cell. In other words, the key was to produce a set of constraints to guide and habilitate the functional activity of other constraints: i.e., a set of encoded, self-referential *meta-*rules (genetic mechanisms).

von Neumann (1966) had the profound intuition that a ‘universal constructor’ must contain its own description, clearly separated from the rest of the system, in order to overcome a fundamental threshold of complexity, from which it can evolve toward other forms of complexity without degradation and decay (see also McMullin, 2000). This fundamental insight, even if it must be reformulated in the light of current biological knowledge (realizing, for instance, that a genome represents a surprisingly minimalist and partial description of the system), and even if it should be properly naturalized (i.e., reconsidered in less abstract/artificial terms: taking the energetic and thermodynamic aspects of the problem into account), remains essentially valid (Ruiz-Mirazo et al., 2008). At its most basic core, life is a long-term sustainable phenomenon on the surface of the Earth because it is capable to propagate reliably the ‘von Neumann architecture’ across space and time (from cell to cell to cell to cell…) thus, avoiding decay. This is why we can assert that life *depends* on ephemeral individuals carrying out their metabolic and cellular activities but it also *transcends* any particular one of them. Somehow, such a fundamental architecture, characteristic of all forms of life, would constitute a ‘super-type’ (or a ‘meta-type’: that is, a type of types that got established during the last stages of the origins of life and has remained there, at its deepest core, ever since).

Returning to our prebiotic context, the relevance of this complex transition in which protocells convert into genetically instructed metabolizing cells, through the development of a translation code, lies in the fact that two huge problems are solved at once: (i) endowing individual non-equilibrium systems with unprecedented dynamic and adaptive robustness, together with (ii) the generation of reliable phylogenies in the population, across time, which mark the beginning of open-ended evolution. Therefore, the first minimal living beings were, indeed, entities with their own identity. First, because they constituted material systems that distinguished themselves from other systems, staying the same individual organization that each of them was, previously in time (in other words, each got realized as a *token* identity). And second, because those material systems were, for all relevant purposes, equivalent to other systems in the (LUCA or ‘pre-LUCA’) population, sharing the same potentialities held by any other member of such a population (i.e., they collectively developed into and, thus, belonged to a *type* identity). Fair enough: one can thus use and build on the concept of identity, starting from the most elementary biological sense. But, turning the question around, what is it that makes this identity actually *biological*? Why is this identity such a special case, intrinsically different from other identities that one may recognize in the physico-chemical world? Let us discuss this key point more carefully and extensively in the next section.

## Core Discussion: the Construction of Biological ‘Inter-Identity’ as the Outcome of a Complex Process of Prebiotic Evolutionary Development

Multiple concurring aspects make the identity of biological entities markedly distinct from other identities that we encounter and try to characterize in the natural world. Most of those aspects derive from the way in which living systems were generated in the first place, and hence the importance of studying the problem in a prebiotic context. Following this genealogical approach, which focuses on the ontological roots and primary causal mechanisms behind the phenomenon, we are now ready to explain that biological identity is singular, among other things, because the processes of construction of *tokens* and *types* get intrinsically interconnected during biogenesis. Then, in the second part of this final section (before concluding with a few general remarks), we will argue that those processes necessarily imply system-system and system-environment interactions of diverse kind, so the use of the idea of ‘inter-identity’ is perfectly justified and, in fact, within the biological domain, it represents a more adequate theoretical choice than bare ‘identity.’

As we have elaborated so far, the origins of life require the development of individuals with a truly convoluted molecular and dynamic organization. Even if one acknowledges the inherent self-organizing properties of matter, in particular if it engages in non-equilibrium states and far-from-trivial transformation processes, the gap between physics/chemistry and biology remains too vast, insurmountable through any clear sequence of steps. Under these circumstances, and despite the many difficulties and uncertainties involved (Ruiz-Mirazo et al., 2017), the road toward biological ‘hyper-complexity’ only seems accessible for heterogeneous protocellular systems (compartmentalized chemical mixtures) that manage to tame spontaneous fission and fusion processes in order to increase their robustness and, at the same time, gain control on their own variability. It is precisely during this transition toward reproductive protocellularity that a new ‘token-type’ relationship emerges in nature. Until that stage, tokens did not have generative power, as far as types were concerned (i.e., the process of coming to existence of each token belonging to a type was, in principle, independent from that of any other particular token within the same type).^8^

However, when protocell fission processes become effectively reproductive, genealogical typologies (‘lineages’) start being produced in the population: namely, (increasingly long) temporal series of protocells that are connected by a continuous line of descent from ancestor to offspring, maintaining similarity across reproductive steps, but bringing about physical discontinuity at each division event, as well (see Figures 3Cii,iii). This spatially and temporally extended set of similar tokens will naturally constitute a type. The more reliable token reproduction becomes, the larger, deeper and more manifest the type will turn out to be, and the more deeply interbred the two (type and token) will get. Now, let us analyze this more carefully (for a similar, complementary view, see Moreno, 2019). A first observation is that the production of these genealogical types is based on the organizational dynamics of individuated tokens, which must recursively grow, reshape their boundary, duplicate their key components and distribute them in such a way that, when fission occurs, two (or more) similar tokens are actually produced. This implies a steady organizational continuity during the process, which is only interrupted when fission occurs. Yet, in order to ensure organizational continuity, the system, strictly speaking, cannot remain the same: it must establish ‘cycles,’ i.e., well-ordered sequences of states in which the progressive creation of an “embryo,” a duplicate of that organization, is naturally integrated within the dynamics of the reproductive token (that is, within the same compartmentalized individual).

A second observation is that the self-reproducing organization triggers an indefinite production of similar –though spatially separate– organizations. Because of this, each individualized entity (token) resulting from a reproductive cycle potentially inherits a specific organizational identity and, when this is actually realized, the sequence of generations constitutes a unique type (lineage). Thus, in an effective reproductive process, there is type continuity, since the mechanism is articulated, precisely, to ensure trans-individual sustainability and, thereby, similarity between the generator and the generated, bringing about an uninterrupted temporal succession of similar organizational tokens. In fact, the latter constitutes the basis of the type continuity between two systems (the reproducer and the reproduced) and, by extension, between all the members of an entire lineage. Again, as the mechanisms of self-production and re-production become more and more robust and reliable, also the degree of similarity between reproducer and reproduced will increase, the propagation process will be extended to larger spatial and temporal scales, and the ‘token-type’ relationship will develop tighter and tighter.

Finally, it should be underlined that these new genealogical types are by no means observer-dependent constructs, but system sets in their own right, endowed with their own causal power. Reproduction establishes an indirect and asymmetric –but deeply relevant– interconnection between the actual token organization and the lineage it contributes and belongs to. Given that the organization of each reproducing protocell triggers a temporal succession of similar entities (namely, the lineage), and given that this concatenated set of similar entities has an important impact at the level of population dynamics (i.e., determining, to a good extent, what stays and what decays), we can say that the type stabilizes that specific form of organization, despite the relatively short duration of the tokens that embody such an organization. In other words, as the reproduction of the organization of particular (transient) tokens becomes increasingly reliable, the type becomes more relevant for the maintenance of that kind of organization, beyond the lifespan of any particular token. Since the same organization is re-generated, once and again, through growth and reproduction, its long-term stabilization comes to depend, ultimately, on that uninterrupted propagation dynamics (which is a fundamental axis of the population dynamics). As a matter of fact, it is this entangled relationship between reproducing protocell/proto-metabolic cycles and their *trans*-generational and causally (spatially and temporarily) more remote consequences, in an environment with limited material resources, that creates the basis for the unfolding of an evolutionary domain. The key point here is to realize that without this domain, which introduces an intrinsic historical dimension in the phenomenon under construction, and whose logics and dynamics cannot be understood unless we resort to a completely new collection of conceptual categories (population, lineage, heredity, selection, fitness function, fitness landscape,…), it is virtually impossible to give a complete account for the origins of full-fledged living individuals.

### On Biological ‘Inter-Identity’

Therefore, it is quite revealing to conceive the construction of biological identity in the context of that process of interbreeding between the physiological-cellular-metabolic ‘token-identity’ of individual, cyclic and reproductive organizations, and the evolutionary-population-historical ‘type-identity’ of collective phylogenies following open-ended (bifurcating and extinguishing) pathways. In that sense, our position is akin to Montévil and Mossio’s (2020), who claim (in this same issue) that biological identity is shaped, in scientific practice, at the crossroads between «historical and relational conceptions [of the living]», carrying out a purely epistemological treatment of the problem. In contrast, we delve into the ontological and genealogical reasons behind the phenomenon, from the point of view of its progressive prebiotic emergence, and this allows us to discuss several issues in greater depth, like the importance of the ‘interactive’ aspects involved – as we do just below, to start concluding our contribution (also summarized schematically in Table 1).

**TABLE 1:** Minimal interactive dimensions required for the construction of biological identity.

Relationship	Interactive processes	Outcome
System-Environment	Matter-energy exchanges	Metabolic organization
System-System (*different* other)	Syntrophic reciprocities	Ecological networks
System-System (*similar* other)	Reproduction/propagation	Phylogenetic pathways

A first fundamental sense in which biological identity conveys interactive processes is related to the non-equilibrium thermodynamic conditions under which any (proto-)metabolic and (proto-)cellular organization must thrive. Biological organisms, together with all their preceding, simpler forms of individuality, beginning from the first relevant self-organizing and self-assembling phenomena (as described in section “A Plausible Departure Point: The ‘Heterogeneous Protocell Population Scenario”’), are necessarily open systems that require the management of matter and energy resources, taken up from the environment, in order to achieve their own, autonomous construction (Ruiz-Mirazo and Moreno, 2004). Just by itself, this dynamic and asymmetric ‘system-environment’ relationship would be enough to argue that the identity of any living being is, in reality, an identity constructed in interaction, or an *inter-identity*. In fact, the capacity of biological systems to modulate functionally that intrinsic and unavoidable coupling they need to maintain with their local environment has developed into multiple and highly sophisticated forms of ‘agency’ (Barandiaran et al., 2009), including the active modification of (more global) boundary conditions in their own benefit. Nevertheless, this is only the basics, the primary stratum on which many other layers and modalities of inter-active dynamic behavior get supported.

A second line of argument to state that any biological identity is intrinsically interactive has to do with the fact that populations of living organisms, right from their very beginning, must be *ecologically* organized. Lacking space here to analyze this topic in sufficient detail, we simply mentioned above how important ecological relationships are to ensure diversity: i.e., not only intra-cellular functional diversity (at the level of the molecular components of an individual), but also inter-cellular phenotypic diversity (at the wider level of individuals within a population). Although both are crucial to overcome evolutionary bottlenecks, the latter (which properly defines the domain for ecology) involves the need to establish consistent, auxiliary ‘system-system’ (i.e., cell–cell) interactions, in such a way that the whole population (ultimately, the emerging biosphere) is sustained by an underlying structure of sub-populations and a complex network of synchronous relationships of inter-dependence among them (food-webs, syntrophy, commensalism, nutrient exchange, and cross-feeding.).^9^ Without this supporting network, in which individuals of diverse (sub-)populations construct a set of ‘niches’ collectively [i.e., in direct interaction/conjunction with individuals of other (sub-)populations], life as a global-level phenomenon would be much more fragile, much weaker against perturbations in the external boundary conditions, and for sure unable to modify actively those boundary conditions. After all, no living cell can develop its ontogenetic existence in an abiotic environment, but in a confederacy or consortium of metabolic reciprocities – i.e., in the context of an ecological organization (Mori et al., 2016; Smith and Morowitz, 2016). From this perspective, the identity of a living entity would be molded, in a highly relevant biological sense, as well, in terms of those sub-populations and ecological relationships involving members of different types. So we are referring here to those aspects of the identity that are constructed through multiple tensions and complementarities with different (biological) *others*.

Nevertheless, in line with the ideas expressed in the initial part of this last section, the most characteristic sense in which biological identities are, in fact, *inter*-identities is linked to the highly convoluted and extended causal tapestry that living organisms must weave in order to complete the process of biogenesis. No cell in nature ever emerged from scratch: it could not, in prebiotic times, and it cannot, today; a cell always comes from another, phylogenetically related cell (Virchow’s dictum, back to the fore). And when a new cell is born, all of its components and organization come directly from the previous one, whose components and organization come from a previous one, and so on and so forth. So biological identity does not belong, exclusively, to any single living individual. It is a complex, transversal and transgenerational construct, with multiple sides and shades of meaning. This is precisely why it can be useful to show the diverse intricacies involved, highlighting the different scales and dimensions of the problem, synchronic and diachronic, that require integration. Cells exist and get realized as individuals; yet, they cannot come to existence and thrive but in the context of populations of similar cells. The development of proto-cellularity was itself an evolutionary process that involved myriads of metabolizing and reproducing individuals, going through a long and wide history of events. Variation (or, more precisely, *control* on variation) must play a fundamental role in that account, too. However, there is little to do without reliable reproduction, understood as the multiplication and propagation of complex organizations (Kauffman, 2000). In addition, we gave several reasons to believe that that is the way it ought to be for any living world to unfold. Therefore, an important part of the ‘inter’ of biological inter-identity is meant to capture those asynchronous, deep and remote linkages that must be established among similar, in-practice-equivalent (biological) *others*.

## Final Remarks: Exploring the Principles of Evolutionary Systems Biology From a Genealogical Perspective

There are many unknowns and open questions about the sequence of transitions from the messy, colloidal scenario described in section two, toward the much more complex stage outlined in the end of the third one, at the onset of biological evolution, where heterogeneity is also ubiquitous but expressed in much more regular, sophisticated and intertwined forms. In any case, no origins-of-life researcher will doubt that, somehow (sooner or later, but within the actual process of biogenesis), prebiotic systems had to develop into functional reproductive protocells. Our claim, quite distinct from the still mainstream views in the field, is that this step had to take place early, so that there was sufficient time and opportunities, from that point onward, for protocell *systems* evolution (Piedrafita et al., 2017; Shirt-Ediss et al., 2017) to proceed. The appearance of endogenous functionalization and reproductive fission in these first protocells would not necessarily coincide (notice the different endings of the triangle peaks in Figure 2B), but they should join forces soon. In this way, the relative importance of exogenous, environmental factors on protocell dynamics and evolution trajectories would progressively diminish, giving way to endogenous protocell activity as the main driver of the process. Nevertheless, as we argued above, many properties emerging in the protocell population, even at the individual level, cannot be accounted for just in purely physiological terms: we need to expand the explanatory context and our repertoire of epistemological constructs to cover wider and longer-term scales, because at least part of the relevant mechanisms (natural selection, ecological niche-construction, genetic drift, geographic distribution, phylogenetic relationships, etc.) operate at those scales.

We consider that this comprehensive prebiotic perspective, which acknowledges the importance of both organizational and evolutionary aspects in the problem, provides a great opportunity to open an investigation program on the fundamental principles underlying biological phenomenology. Living systems are hyper-complex, indeed, and facing that complexity upfront, all at once, is extremely hard. Multiple decomposing or simplifying strategies have been tried during the –still short– history of natural sciences, with diverse degrees of success. Actually, most of what we know about the living domain comes from those analytic strategies, which should continue being pursued and developed further in the future. Nevertheless, complementary integrative approaches must be implemented, as well, like the young field of ‘systems biology’ has already demonstrated (Westerhoff and Palsson, 2004; Boogerd et al., 2007; Hübner et al., 2011). Yet, most of those approaches have been applied, so far, to start filling in the apparent gaps between molecular and cell biology. Perhaps an alternative and potentially very fruitful idea would be to elaborate explanations from the bottom-up, but in a strong *genealogical* sense (i.e., starting from biologically inspired chemistry). Origins-of-life research has the advantage that the relevant systems under scrutiny, by definition, ought to be simpler than living beings: the further back in biogenesis, the simpler they should actually be. In this way, the emergence of increasing layers of complexity during the process, and the general principles behind each transition step can be much more explicitly and precisely addressed (whereas the study of real biological systems forces us to deal with all those –deeply intertwined– layers at the same time).

Systems biology, despite its remarkable advances in recent years, is still awaiting key theoretical insights to unveil the general principles of organization behind life’s complexity (beyond the non-reductionist philosophy and methods developed from network theory and the sciences of self-organization). In addition, several authors have suggested that a new synthesis is required, and has already begun, in which *systems* and *evolutionary* theory merge (Soyer and O’Malley, 2013; O’Malley et al., 2015). Investigations on the origins of life, especially if they contemplate the actual interbreeding between organizational and evolutionary aspects of the problem (e.g., working with various kinds of protocell families, but including in the study short-/long-term effects coming from their population dynamics), could constitute very fertile ground for this ambitious project of bringing together two major traditions in the life sciences (the physiological and evolutionary traditions), and try to generalize, thereafter, biological theory. One may even dare to say that those investigations represent the most promising avenue of research in that direction, with an important input from the currently flourishing field of ‘systems chemistry’ (Ruiz-Mirazo et al., 2014, 2017).

In any case, the magnitude of the challenge ahead should not be underestimated. We, as human beings (and more so as scientists), tend to search for shortcuts, for simplified pathways that logically connect different phenomena and observations of the world surrounding us. This is just our natural way of learning and understanding. So those of us especially interested in the advent of living cells are, of course, eager to learn and understand the process of biogenesis before our own cells cease to exist. Yet, the complex tapestry of life must be autonomously weaved… and deciphering all the inter-identities involved may take quite a bit of time, effort and patience.

## Author Contributions

KR-M and AM conceived initially the work and the main line of argument. All authors took part in subsequent discussions and contributed with constructive feedback and suggestions for improvement. KR-M wrote a first draft of the manuscript. BS-E produced the figures, in close interaction with KR-M. ME-C completed the list of references and took care of the submission procedure.

## Conflict of Interest

The authors declare that the research was conducted in the absence of any commercial or financial relationships that could be construed as a potential conflict of interest.

## Footnotes

1 Not only from endogenous sources (Rushdi and Simoneit, 2001; McCollom and Seewald, 2006), but also coming from meteorites and additional extra-terrestrial processes/bodies (Deamer, 1986; Dworkin et al., 2001).

2 Other authors have previously suggested the relevance of a similar scenario for the origins of life (e.g., Norris and Raine, 1998), but we will explore here several specific aspects and non-trivial implications of it, which have not been considered in the literature so far.

3 In fact, as we will discuss in more detail below, in such a scenario it is difficult to characterize those chemically reacting compartmentalized systems as tokens whose similarities could derive into stable types. At least, according to a concept of ‘type’ that assumes the ontological pre-existence of well-identifiable ‘tokens’ that share a number of similarities. One could, of course, try to isolate and characterize embryonic individuals, emerging out of the myriads of interactions within that global colloidal messiness; and, then, try to classify those diverse members of the protocell population into several groups (according to different criteria: e.g., vesicle size and composition are the most obvious – but also their shape, individual mass density, internal concentration of organic matter, their propensity to form clusters, their growth kinetics, the osmotic tension of their membrane, etc.). However, the continuous, highly stochastic transformation of the complex colloid would make this task, in practice, impossible.

4 Exponential growth rates lead to mutually exclusive situations (there has to be a winner), whereas sub-exponential growth rates (e.g., parabolic ones, for instance) allow for co-existence of multiple types in the population (Szathmáry and Gladkih, 1989). The mechanism of natural selection (NS) has been traditionally linked to exponential growth dynamics, but this remains a property of highly idealized evolutionary models. In a more realistic setting, like the prebiotic scenario proposed in this paper, we consider that diverse selective pressures would be operating at different levels (molecular, proto-cellular, proto-ecological…) and across various temporal scales. Those pressures and selection dynamics ought to be properly characterized in future research on prebiotic evolution, including the analysis of their relative strengths (not only in terms of mean growth/reproduction rates of the individuals within a population at a certain stage, as it is classically done, but also throughout consecutive generations, in wider time windows – e.g., in terms of ‘individual histories’; Leibler and Kussell, 2010).

5 Thus, the notion of *type* gains ontological status at this stage. It is not just an epistemological option, because the protocell population gets actually distributed in ‘families’ or ‘breeds’ and any collective description of the global system state must capture these manifest asymmetries (see Figure 2). But the most interesting –and somewhat paradoxical– point here is that *reproduction (the generation of a type) is what stabilizes the token identity of the reproducing protocells.* Thus, once again, it is through the interactions (here, through the uninterrupted sequence of reproductive cycles) that a more robust form of self-constructing token identity appears. As we have seen, the previous situation depicts a world of unstable, ever-changing compartmentalized systems. Yet, when some protocells are endowed with the intrinsic capacity to produce –both spatially and temporally– similar entities, they will stabilize a specific kind of token. Obviously, not the same token; but, rather, *its identity as a token*.

6 Given the far-from-equilibrium conditions under which these systems had to operate, together with the continuous processes of fission and fusion going on in the population, the game obviously is not played just *within* individual protocells but also *among* them (see section “Core Discussion: The Construction of Biological ‘Inter-identity’ as the Outcome of a Complex Process of Prebiotic Evolutionary Development”).

7 As we commented in previous paragraphs, phenotypic diversity had to be present in any ‘pre-LUCA’ cell population, so narrowness here is a relative notion, of course. Yet, the amount of basic material and organizational features shared among the members of that population had to be exceptional, leading to a global picture of strong homogeneity, in comparison with previous phases (see upper part of Figure 2), and especially regarding the “chassis” of the cells (the characteristic way of organizing their fundamental components and transformation processes).

8 Perhaps there could be some discussion on whether molecules that are synthesized through autocatalytic mechanisms, which are widely spread in chemistry, contradict this statement… but, since a seed is always required for autocatalysis to proceed, an alternative pathway for the primary production of those molecules should be available, at any rate. In contrast, this is definitely not the case when we speak about living cells (recall Virchow’s dictum: *omnis cellula ex cellula*).

9 A word of caution here about ‘synchronicity’: ecological time scales are, of course, longer than the characteristic physiological (ontogenetic) ones, since ecosystems involve changes at the level of populations (or sub-populations) and beyond. However, in comparison with intrinsically asynchronous relationships, like phylogenetic connections, it is quite clear that ecological mechanisms and organizations should be conceived as synchronous, in the sense that they operate at the same time scales as the actual population/sub-population dynamics.

## References

[B1] Alberts B. Johnson A. Lewis J. Raff M. Roberts K. Walter P. (2002). *Molecular Biology of the Cell.* New York, NY: Garland Science.

[B2] Barandiaran X. Di Paolo E. Rohde M. (2009). Defining agency: individuality, normativity, asymmetry, and spatio-temporality in action. *Adapt. Behav.* 17 367–386. 10.1177/1059712309343819

[B3] Bickhard M. H. (2000). Autonomy, function, and representation. *Commun. Cogn. Artif. Intellig.* 17 111–131.

[B4] Bishop R. C. (2012). Fluid convection, constraint and causation. *Interface Focus* 2 4–12. 10.1098/rsfs.2011.0065 23386955 PMC3262306

[B5] Boogerd F. C. Bruggeman F. J. Hofmeyr J.-H. S. Westerhoff H. V. (eds) (2007). *Systems Biology. Philosophical Foundations.* Amsterdam: Elsevier Science.

[B6] Brubaker R. Cooper F. (2000). Beyond ‘identity’. *Theor. Soc.* 29 1–47. 10.1186/s12960-018-0338-0 30616656 PMC6323796

[B7] Carrara P. Stano P. Luisi P. L. (2012). Giant Vesicles “Colonies”: a model for primitive cell communities. *ChemBioChem* 13 1497–1502. 10.1002/cbic.201200133 22689306

[B8] Collier J. D. Hooker C. A. (1999). Complexly organised dynamical systems. *Open Syst. Inform. Dyn.* 6:241. 10.1023/A:1009662321079 16049729

[B9] Deamer D. W. (1986). Role of amphiphilic compounds in the evolution of membrane structure on the early earth. *Orig. Life Evol. Biosph.* 17 3–25. 10.1007/BF01809809 3796965

[B10] Dworkin J. P. Deamer D. W. Sandford S. A. Allamandola L. J. (2001). Self-assembling amphiphilic molecules: synthesis in simulated interstellar/precometary ices. *Proc. Natl. Acad. Sci. U.S.A.* 98 815–819. 10.1073/pnas.98.3.815 11158552 PMC14665

[B11] Gogarten J. P. Deamer D. W. (2016). Is LUCA a thermophilic progenote? *Nat. Microbiol.* 1:16229 10.1038/nmicrobiol.2016.229 27886195

[B12] Guerrero R. (1995). “Vida arcaica y ecopoyesis,” in *Los Orígenes de la Vida*, eds Morán F. Peretó J. Moreno A. (Madrid: Editorial Complutense), 225–243.

[B13] Guerrero R. (1998). Crucial crises in biology: life in the deep biosphere. *Int. Microbiol.* 1 285–294. 10943376

[B14] Hübner K. Sahle S. Kummer U. (2011). Applications and trends in systems biology in biochemistry. *FEBS J.* 278 2767–2857. 10.1111/j.1742-4658.2011.08217.x 21707921

[B15] Javaux E. J. (2019). Challenges in evidencing the earliest traces of life. *Nature* 572 451–460. 10.1038/s41586-019-1436-4 31435057

[B16] Juarrero A. (2002). Complex dynamical systems and the problem of identity. *Emergence* 4 94–104. 10.emerg/10.17357.a06c091343f9fc23c466317046f0881d

[B17] Kauffman S. (2000). *Investigations.* Oxford: Oxford University Press.

[B18] Kroiss D. Ashkenasy G. Braunschweig A. B. Tuttle T. Ulijn R. V. (2019). Catalyst: can systems chemistry unravel the mysteries of the chemical origins of life? *Chemistry* 5 1917–1920. 10.1016/j.chempr.2019.05.003

[B19] Kurisu M. Aoki H. Jimbo T. Sakuma Y. Imai M. Serrano-Luginbühl S. (2019). Reproduction of vesicles coupled with a vesicle surface-confined enzymatic polymerisation. *Commun. Chem.* 2:117 10.1038/s42004-019-0218-0

[B20] Leibler S. Kussell E. (2010). Individual histories and selection in heterogeneous populations. *Proc. Natl. Acad. Sci. U.S.A.* 107 13183–13188. 10.1073/pnas 20616073 PMC2919897

[B21] Maturana H. Varela F. (1980). *Autopoiesis and Cognition: The Realization of the Living.* Dordrecht: Springer.

[B22] Mavelli F. Ruiz-Mirazo K. (2013). Theoretical conditions for the stationary reproduction of model protocells. *Integr. Biol.* 5 324–341. 10.1039/c2ib20222k 23233152

[B23] McCollom T. M. Seewald J. S. (2006). Carbon isotope composition of organic compounds produced by abiotic synthesis under hydrothermal conditions. *Earth Planet. Sci. Lett.* 243:74–84.

[B24] McMullin B. (2000). John von Neumann and the evolutionary growth of complexity: looking backwards, looking forwards. *Artif. Life* 6 347–361. 10.1162/106454600300103674 11348586

[B25] Montévil M. Mossio M. (2020). The identity of organisms in scientific practice: integrating historical and relational conceptions [this issue]. *Front. Physol.* 10.3389/fphys.2020.00611 PMC7311753 32625111

[B26] Moreno A. (2019). The origin of a trans-generational organization in the phenomenon of biogenesis. *Front. Physiol.* 10:1222. 10.3389/fphys.2019.01222 31611810 PMC6769072

[B27] Moreno A. Ruiz-Mirazo K. (2009). The problem of the emergence of functional diversity in prebiotic evolution. *Biol. Philos.* 24 585–605. 10.1007/s10539-009-9178-6

[B28] Mori M. Ponce-de-León M. Peretó J. Montero F. (2016). Metabolic complementation in bacterial communities: necessary conditions and optimality. *Front. Microbiol.* 7:1553. 10.3389/fmicb.2016.01553 27774085 PMC5054487

[B29] Nelson D. L. Cox M. (2017). *Lehninger Principles of Biochemistry*, 7th Edn New York: W.H.Freeman & Co Ltd.

[B30] Norris V. Raine D. J. (1998). A fission-fusion origin for life. *Orig. Life Evol. Biosph.* 28 523–537. 10.1023/a:1006568226145 9742727

[B31] Oglêcka K. Rangamani P. Liedberg B. Kraut R. S. Parikh A. N. (2014). Oscillatory phase separation in giant lipid vesicles induced by transmembrane osmotic differentials. *eLife* 3:e03695. 10.7554/eLife.03695 25318069 PMC4197780

[B32] O’Malley M. A. Soyer O. S. Siegal M. L. (2015). A philosophical perspective on evolutionary systems biology. *Biol. Theory* 10 6–17. 10.1007/s13752-015-0202-6 26085823 PMC4465572

[B33] Pattee H. H. (1969). “How does a molecule become a message?,” in *Communication in Development*, Vol. 3 ed. Lang A. (New York, NY: Academic Press), 1–16.

[B34] Pattee H. H. (1977). Dynamic and linguistic modes of complex systems. *Int. J. Gen. Syst.* 3 259–266.

[B35] Piedrafita G. Monnard P.-A. Mavelli F. Ruiz-Mirazo K. (2017). Permeability-driven selection in a semi-empirical protocell model: the roots of prebiotic systems evolution. *Sci. Rep.* 7:3141. 10.1038/s41598-017-02799-6 28600550 PMC5466667

[B36] Prigogine I. (1980). *From being to becoming: time and complexity in the physical sciences.* New York, NY: Freeman & Co.

[B37] Ruiz-Mirazo K. (2011). “Protocell,” in *Encyclopedia of Astrobiology*, eds Gargaud M. Amils R. Cernicharo Quintanilla J. Cleaves H. J. Irvine W. M. Pinti D. (Heidelberg: Springer), 1353–1354.

[B38] Ruiz-Mirazo K. (2019). Reaction: a plea for hypothesis-driven research in prebiotic systems chemistry. *Chemistry* 5 1920–1922. 10.1016/j.chempr.2019.06.009

[B39] Ruiz-Mirazo K. Briones C. de la Escosura A. (2014). Prebiotic systems chemistry: new perspectives for the origins of life. *Chem. Rev.* 114 285–366. 10.1021/cr2004844 24171674

[B40] Ruiz-Mirazo K. Briones C. de la Escosura A. (2017). Chemical roots of biological evolution: the origins of life as a process of development of autonomous functional systems. *Open Biol.* 7:170050. 10.1098/rsob.170050 28446711 PMC5413913

[B41] Ruiz-Mirazo K. Mavelli F. (2008). On the way towards ‘basic autonomous agents’: stochastic simulations of minimal lipid–peptide cells. *Biosystems* 91 374–387. 10.1016/j.biosystems.2007.05.013 17714858

[B42] Ruiz-Mirazo K. Moreno A. (2004). Basic autonomy as a fundamental step in the synthesis of life. *Artif. Life* 10 235–259. 10.1162/1064546041255584 15245626

[B43] Ruiz-Mirazo K. Umerez J. Moreno A. (2008). Enabling conditions for ‘open-ended evolution’. *Biol. Philos.* 23 67–85.

[B44] Rushdi A. I. Simoneit B. R. (2001). Lipid formation by aqueous Fischer-Tropsch-type synthesis over a temperature range of 100 to 400 degrees C. *Orig. Life Evol. Biosph.* 31 103–118. 10.1023/a:1006702503954 11296515

[B45] Shirt-Ediss B. (2016). Modelling early transitions toward autonomous protocells. *ArXiv [Preprint].* Available online at: https://arxiv.org/abs/1606.03620 (accessed May 13, 2020).

[B46] Shirt-Ediss B. Murillo-Sánchez S. Ruiz-Mirazo K. (2017). Framing major prebiotic transitions as stages of protocell development: three challenges for origins-of-life research. *Beilstein J. Organ. Chem.* 13 1388–1395. 10.3762/bjoc.13.135 28781704 PMC5530630

[B47] Shirt-Ediss B. Ruiz-Mirazo K. Mavelli F. Solé R. V. (2014). Modelling lipid competition dynamics in heterogeneous protocell populations. *Sci. Rep.* 4:5675. 10.1038/srep05675 25024020 PMC4097352

[B48] Shirt-Ediss B. Solé R. Ruiz-Mirazo K. (2015). Emergent chemical behavior in variable-volume protocells. *Life* 5 181–211. 10.3390/life5010181 25590570 PMC4390847

[B49] Simon H. A. (1962). The architecture of complexity. *Proc. Am. Philos. Soc.* 106 467–482.

[B50] Smith E. Morowitz H. J. (2016). *The Origin and Nature of Life on Earth: The Emergence of the Fourth Geosphere.* Cambridge: Cambridge University Press.

[B51] Souza T. P. Stano P. Luisi P. L. (2009). The minimal size of liposome-based model cells brings about a remarkably enhanced entrapment and protein synthesis. *ChemBioChem* 10 1056–1063. 10.1002/cbic.200800810 19263449

[B52] Souza T. P. Steiniger F. Stano P. Fahr A. Luisi P. L. (2011). Spontaneous crowding of ribosomes and proteins inside vesicles: a possible mechanism for the origin of cell metabolism. *ChemBioChem* 12 2325–2330. 10.1002/cbic.201100306 21830290

[B53] Soyer O. S. O’Malley M. A. (2013). Evolutionary systems biology: what it is and why it matters. *Bioessays* 35 696–705. 10.1002/bies.201300029 23681824

[B54] Stryer L. Berg J. M. Tymoczko J. L. Gatto G. J. (2015). *Biochemistry*, 8th Edn New York, NY: Palgrave Macmillan.

[B55] Sutherland J. D. (2017). Opinion: studies on the origin of life — the end of the beginning. *Nat. Rev. Chem.* 1:0012 10.1038/s41570-016-12

[B56] Szathmáry E. Gladkih I. (1989). Sub-exponential growth and coexistence of non-enzymatically replicating templates. *J. Theor. Biol.* 138 55–58. 10.1016/s0022-5193(89)80177-8 2483243

[B57] von Neumann J. (1966). *Theory of self-Reproducing Automata*, ed. Burks A. W. (Urbana: The University of Illinois Press).

[B58] Weiss M. C. Preiner M. Xavier J. C. Zimorski V. Martin W. F. (2018). The last universal common ancestor between ancient Earth chemistry and the onset of genetics. *PLoS Genet.* 14:e1007518. 10.1371/journal.pgen.1007518 30114187 PMC6095482

[B59] Westerhoff H. V. Palsson B. O. (2004). The evolution of molecular biology into systems biology. *Nat. Biotechnol.* 22 1249–1252. 10.1038/nbt1020 15470464

[B60] Woese C. R. (1998). The universal ancestor. *PNAS* 95 6854–6859. 10.1073/pnas.95.12.6854 9618502 PMC22660

[B61] Woese C. R. Fox G. E. (1977). Phylogenetic structure of the prokaryotic domain: the primary kingdoms. *Proc. Natl. Acad. Sci. U.S.A.* 74 5088–5090. 10.1073/pnas.74.11.5088 270744 PMC432104

[B62] Wong J. T. (1975). A co-evolution theory of the genetic code. *Proc. Natl. Acad. Sci. U.S.A.* 72 1909–1912. 10.1073/pnas.72.5.1909 1057181 PMC432657

[B63] Wong J. T. Ng S.-K. Mat W.-K. Hu T. Xue H. (2016). Coevolution theory of the genetic code at age forty: pathway to translation and synthetic life. *Life* 6:12. 10.3390/life6010012 26999216 PMC4810243

[B64] Xavier J. C. Patil K. R. Rocha I. (2014). Systems biology perspectives on minimal and simpler cells. *Microbiol. Mol. Biol. Rev.* 78 487–509. 10.1128/MMBR.00050-13 25184563 PMC4187685

[B65] Zepik H. H. Blöchliger E. Luisi P. L. (2001). A chemical model of homeostasis. *Angewandte Chem.* 113 205–208. 10.1002/1521-3773(20010105)40:1<199::AID-ANIE199>3.0.CO;2-H 29711953

